# Artificial Intelligence-Assisted Comparative Analysis of the Overlapping Molecular Pathophysiology of Alzheimer’s Disease, Amyotrophic Lateral Sclerosis, and Frontotemporal Dementia

**DOI:** 10.3390/ijms252413450

**Published:** 2024-12-15

**Authors:** Zihan Wei, Meghna R. Iyer, Benjamin Zhao, Jennifer Deng, Cassie S. Mitchell

**Affiliations:** 1 Laboratory for Pathology Dynamics, Department of Biomedical Engineering, Georgia Institute of Technology & Emory University School of Medicine, Atlanta, GA 30322, USA; 2 Center for Machine Learning at Georgia Tech, Atlanta, GA 30332, USA

**Keywords:** Alzheimer’s disease (AD), amyotrophic lateral sclerosis (ALS), frontotemporal dementia (FTD), neuropathology, machine learning, network regulation, literature-based discovery, knowledge graph, neurophysiology, pathology dynamics

## Abstract

The overlapping molecular pathophysiology of Alzheimer’s Disease (AD), Amyotrophic Lateral Sclerosis (ALS), and Frontotemporal Dementia (FTD) was analyzed using relationships from a knowledge graph of 33+ million biomedical journal articles. The unsupervised learning rank aggregation algorithm from SemNet 2.0 compared the most important amino acid, peptide, and protein (AAPP) nodes connected to AD, ALS, or FTD. FTD shared 99.9% of its nodes with ALS and AD; AD shared 64.2% of its nodes with FTD and ALS; and ALS shared 68.3% of its nodes with AD and FTD. The results were validated and mapped to functional biological processes using supervised human supervision and an external large language model. The overall percentages of mapped intersecting biological processes were as follows: inflammation and immune response, 19%; synapse and neurotransmission, 19%; cell cycle, 15%; protein aggregation, 12%; membrane regulation, 11%; stress response and regulation, 9%; and gene regulation, 4%. Once normalized for node count, biological mappings for cell cycle regulation and stress response were more prominent in the intersection of AD and FTD. Protein aggregation, gene regulation, and energetics were more prominent in the intersection of ALS and FTD. Synapse and neurotransmission, membrane regulation, and inflammation and immune response were greater at the intersection of AD and ALS. Given the extensive molecular pathophysiology overlap, small differences in regulation, genetic, or environmental factors likely shape the underlying expressed disease phenotype. The results help prioritize testable hypotheses for future clinical or experimental research.

## 1. Introduction

Amyotrophic Lateral Sclerosis (ALS), Alzheimer’s Disease (AD), and Frontotemporal Dementia (FTD) are typically described as distinct neurodegenerative diseases with overlapping molecular features and, in some cases, co-occurrence. Each disease has characteristic clinical manifestations: ALS primarily affects motor neurons, leading to muscle weakness and paralysis; AD causes memory loss and cognitive decline due to degeneration in the hippocampus and cerebral cortex; and FTD results in personality, behavior, and language changes due to frontal and temporal lobe degeneration [1]. Despite these differences, ALS, AD, and FTD share common molecular pathophysiologies that contribute to overlapping and occasionally concurrent disease presentations [1,2,3].

One major link among these diseases is protein aggregation, a common feature across neurodegenerative disorders. ALS is marked by aggregates of proteins like TDP-43, SOD1, and FUS in motor neurons, which disrupt cellular function [4]. Similarly, in AD, amyloid-beta plaques and tau tangles accumulate in the brain, particularly in memory-related regions. FTD can involve tau or TDP-43 protein pathology, depending on the subtype, leading to degeneration in the frontal and temporal regions. TDP-43 proteinopathy is particularly significant as it serves as a common denominator in ALS, many FTD cases, and some AD cases, promoting neuronal loss and linking ALS-FTD as a combined disorder [5].

Genetic mutations also connect these diseases [6], particularly the C9orf72 gene, which is associated with both ALS and FTD. Expansions in *C9orf72* lead to RNA toxicity and dipeptide repeat proteins that disrupt cellular processes, explaining why patients with this mutation may exhibit symptoms of both ALS and FTD [7]. Mutations in other genes, such as TARDBP (encoding TDP-43) and MAPT (encoding tau), further illustrate genetic overlap [8]. For example, tau mutations are common in FTD and may also contribute to AD pathology.

Mitochondrial dysfunction and oxidative stress are additional overlapping mechanisms. ALS motor neurons are particularly vulnerable to mitochondrial stress, leading to energy deficits and cell death [9]. In AD, oxidative stress exacerbates amyloid-beta toxicity and tau pathology, impairing energy production in neurons [10]. FTD also displays mitochondrial impairment, especially in TDP-43 or tau-based forms, [11] linking these energy-related deficits across the diseases.

Neuroinflammation is another shared pathway. Microglial and astrocytic activation occurs in response to protein aggregates and neuronal damage, releasing inflammatory cytokines that further promote neurodegeneration. In AD, amyloid plaques trigger chronic neuroinflammation, damaging surrounding neurons [12]. Similarly, ALS involves inflammatory responses around degenerating motor neurons, and FTD exhibits inflammation in affected cortical areas [13]. This neuroinflammatory environment, common to ALS, AD, and FTD, accelerates disease progression.

Co-occurrences such as ALS-FTD, AD-FTD, and ALS-AD, though rare, exemplify the spectrum of neurodegeneration, where shared pathophysiological pathways lead to overlapping clinical and molecular characteristics. ALS-FTD is the most common overlap or co-diagnosis; it is often linked to TDP-43 pathology and *C9orf72* gene mutations [7,14]. Up to 15% of ALS patients develop clinical signs of FTD, while about 50% show some degree of cognitive or behavioral impairment associated with FTD [14,15]. Conversely, a smaller subset of FTD patients may develop motor neuron symptoms characteristic of ALS. AD-FTD co-diagnosis, often referred to as “mixed dementia” due to the presence of cognitive and behavior symptomatology, is less frequent [16]; it involves the presence of both tau and amyloid-beta pathology associated with AD, along with TDP-43 or tau pathologies characteristic of FTD. ALS-AD, which is the most rare combination [17], may occur due to shared vulnerabilities in mitochondrial and inflammatory pathways that increase neurodegenerative susceptibility.

The overlapping clinical manifestations and molecular pathophysiologies suggest that ALS, AD, and FTD may not be entirely separate diseases. Rather, they may be part of a neurodegenerative continuum [18] or a neurodegenerative spectrum. Understanding the shared molecular mechanisms underlying neurodegeneration provides valuable insights into the spectrum of these disorders and may facilitate the development of therapies targeting common pathological pathways.

To this end, the present study employed artificial intelligence-driven methodologies to explore, prioritize, and quantify the degree of overlapping molecular pathophysiology between AD, ALS, and FTD. Artificial intelligence facilitates the synthesis of knowledge with a depth and breadth of comprehensive data sources not possible as part of a traditional manual systematic review. Moreover, AI algorithms can alleviate bias caused by siloed information in specialized domains [19]. Specifically, AI can analyze multi-factorial and multi-scalar relationships from millions of journal articles, facilitating the synthesis of knowledge in a comprehensive and less biased manner [20,21]. In contrast, traditional systematic reviews typically encompass only a small fraction of narrowly defined articles, which typically range from 100 to 200 articles [8] and often overlook cross-domain connections.

The present study leveraged 33+ million journal articles to construct and query a semantic knowledge graph, SemNet 2.0 [21], to examine the literature-defined overlapping molecular concepts (nodes) and relationships (edges) between AD, ALS, and FTD. Metapaths define the arrangement of relationships between user-defined biomedical concepts of interest, termed as target nodes, and related biomedical concepts within the graph, known as source nodes. An unsupervised ranking algorithm was employed to determine the most important intersecting molecular pathophysiology concepts (source nodes), using a relevance-based metric called a HeteSim score [21]. Finally, human-in-the-loop supervision [22,23] and supervised large language model evaluation provided a means of external validation [19].

## 2. Results and Discussion

The presented results of this study quantify the degree of molecular pathophysiology overlap between AD, ALS, and FTD using data from 33+ million journal articles and a combination of unsupervised and supervised artificial intelligence-assisted approaches. First, metadata from the list of SemNet 2.0 simulations is examined to explore the number of intersecting source nodes returned that are shared by disease pairs. Second, the Unified Medical Language System (UMLS) node type of amino acids, peptides, proteins (AAPP) —which represents sub-molecular and molecular pathophysiology—was analyzed to rank the most important nodes to AD, ALS, or FTD. Source nodes shared between or “intersecting” disease pairs were examined to better elucidate molecular pathophysiological overlap. Finally, biological processes were mapped to enable a more comprehensive comparison of their relative contributions to the shared molecular pathophysiology underlying overlapping neurodegenerative disease mechanisms.

### 2.1. Analysis of Metadata

Table 1 summarizes the source node count output from SemNet 2.0 simulations for each disease (AD, FTD, ALS) for the AAPP node type. Overall, AD had the most returned related source nodes, while FTD had the least number of returned related source nodes. The number of identified source nodes is a function of the SemNet 2.0 search parameters as well as the number of literature sources for each target disease (AD, ALS, FTD). In the present study, the SemNet 2.0 search parameters were the same for each disease. Thus, any differences in the number of returned related source nodes was a function of the number of literature data sources attributed to the specified target disease.

Table 2 summarizes the numbers of intersections of every two diseases and the union of all three diseases. The counts of source nodes shared by each disease pair provide an estimate of the degree of shared physiology between the diseases based on extracted literature relationships. The number of shared nodes provides evidence to support the possible presence of a pathophysiological disease spectrum.

### 2.2. Analysis of AAPP Overlap in AD, ALS, and FTD

**Figure 1:**
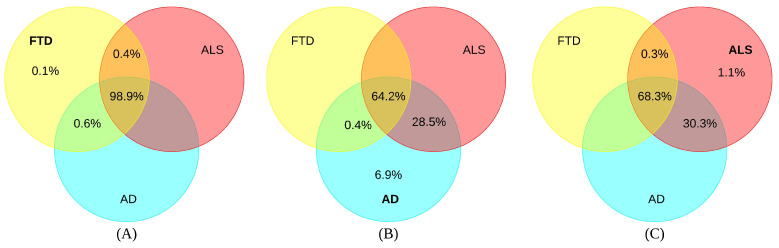
A Venn diagram of three diseases (AD, ALS, and FTD) illustrating intersections and unions for AAPP (amino acid, peptide, proteins) node type. FTD is represented by the light yellow circles; AD is represented by the aqua blue circles; ALS is represented by the light red circles. Intersections are shown in percentages for each disease. (**A**) FTD; (**B**) AD; (**C**) ALS.

Venn diagrams were used to compare and contrast overlapping nodes between disease pairs as a percentage. In Figure 1A, 98.9% of all AAPP source nodes in FTD are in the union of all three diseases. 0.4% of these nodes in FTD are shared with ALS, and 0.6% are shared with AD. Only 0.1% of these nodes are exclusively related to FTD. In Figure 1B, 64.2% of all AAPP source nodes in AD are in the union of all three diseases. Additionally, 28.5% of these nodes in AD are shared with ALS, and 0.4% are shared with FTD. A toal of 6.9% of nodes are exclusively related to AD. In Figure 1C, 68.3% of all AAPP source nodes in ALS are in the union of all three diseases. Further, 0.3% of these nodes in ALS are shared with FTD, and 30.3% are shared with AD. In total, 1.1% of nodes are exclusively related to ALS.

**Figure 2:**
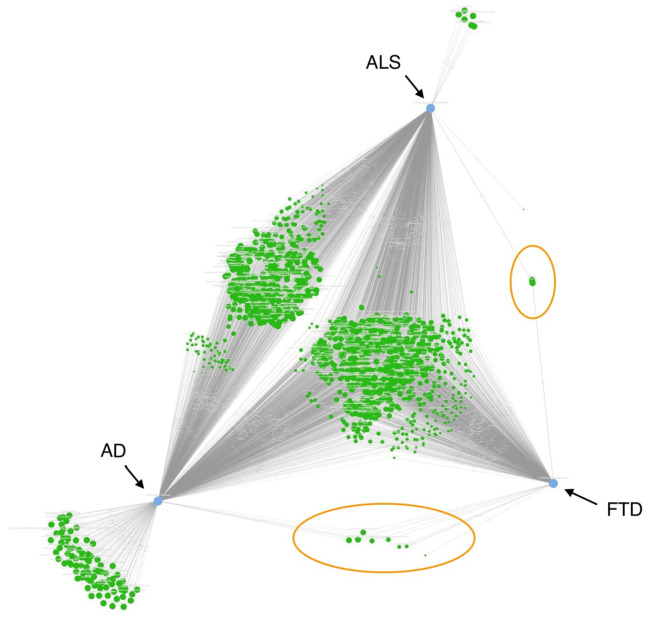
CompositeView of top 1% AAPP (amino acid peptide protein) nodes from AD, ALS, and FTD. CompositeView is a visualization tool for SemNet 2.0 that allows for the aggregation and visualization of multiple simulation outputs into a compressed form [24]. The source nodes are colored in green, while the target nodes are colored in blue with names next to them. There are large amounts of source nodes shared only between AD and ALS; meanwhile, the AD and FTD and ALS and FTD disease pairs (circled in orange) fewer nodes. The majority of nodes are shared by all three diseases. Nodes exclusive to only one disease are labeled outside the apex: AD has many exclusive nodes; ALS has a few exclusive nodes; and FTD had no exclusive nodes.

The UMLS AAPP node type has a large volume of source nodes, and its inherent diversity makes it a complex node type. Figure 2 shows the overall view produced by CompositeView to visualize the connections of the top 1% of AAPP source nodes between the target diseases of AD (left), ALS (top), and FTD (right). CompositeView is a visualization tool for SemNet 2.0 that allows the aggregation and visualization of multiple simulation outputs in a compressed form [24]. The nodes that project to the triangular center represent the AAPP nodes shared by all three diseases.

### 2.3. Mapping Biological Processes to Holistically Examine Disease Spectrum Overlap

**Figure 3:**
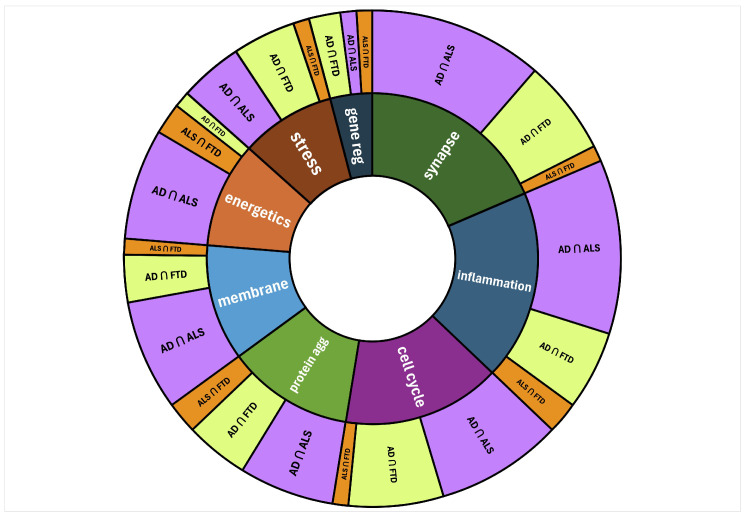
Sunburst diagram of high-ranking AAPP nodes for each disease intersection mapped to their biological processes. The width of each segment represents the number of source nodes. The inner ring shows the relative counts of high-ranking intersecting nodes belonging to each biological process. Clockwise from the top of the inner circle: synapse and neurotransmission (dark green); inflammation and immune response (navy); cell cycle regulation (dark purple); protein aggregation (green); membrane regulation (turquoise); energetics and metabolism (dark orange); stress response regulation (brown); gene regulation and expression (obsidian). The outer ring shows the relative counts of the mapped biological processes for each disease intersection: AD ∩ ALS, AD ∩ FTD, ALS ∩ FTD.

Every top-ranked AAPP node was assigned to one or more of predefined biological processes to better understand the differences in functional etiology between diseases. Each node was assigned to one or more of eight biological processes. The occurrence of predefined biological processes was counted to understand their relative contribution for each intersecting disease pair. The predefined biological processes are shown in different colors in the inner ring of Figure 3: synapse and neurotransmission; inflammation and immune response; cell cycle regulation; protein aggregation; membrane regulation; energetics and metabolism; stress response regulation; and gene regulation and expression.

The inner ring (Figure 3) is a visual examination of the relative weight of each biological process based solely on the top 1% of high-ranking nodes. The outer ring segments of Figure 3 examined how much the intersection of each pair of diseases contributed to each biological process. However, this figure depicted total counts that had not yet been normalized by the number of nodes for each disease intersection. The AD and ALS simulations and the corresponding intersection contain the most nodes. Thus, the outer ring segments were typically largest for AD ∩ ALS.

**Figure 4:**
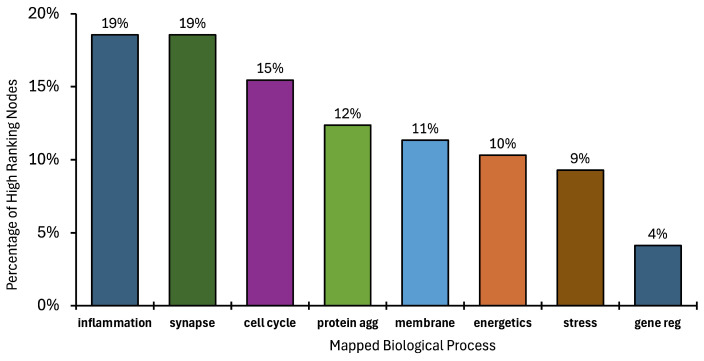
Bar chart quantitatively illustrating the biological mapping of the top 1% of high-ranking AAPP nodes based on all disease intersections: AD ∩ ALS, AD ∩ FTD, and ALS ∩ FTD.

Figure 4 shows the percentage of mapped biological processes for the top 1% of intersecting nodes for AD, ALS, and FTD: inflammation and immune response, 19%; synapses and neurotransmission, 19%; cell cycle, 15%; protein aggregation, 12%; membrane regulation, 11%; stress response and regulation, 9%; gene regulation, 4%. Note that these percentages align with the relative weights of the inner ring of Figure 3.

The hierarchical sunburst diagram in Figure 3 shows the relevance between each disease pair and the overall weight of the biological processes. Comparison of the biological processes between intersecting diseases yields some interesting findings. Inflammation and immune response was more represented in the intersection of AD with ALS but was less represented in the intersection of AD and FTD. High-ranking AAPP nodes representing brain and neuronal development were more prevalent in the intersection of AD and FTD than the intersection of AD and ALS. Energy metabolism, namely lipid metabolism, was more relevant to the intersection of AD and FTD. Synaptic transmission and signaling was important to both the intersection of AD and ALS and the intersection of AD and FTD.

**Figure 5:**
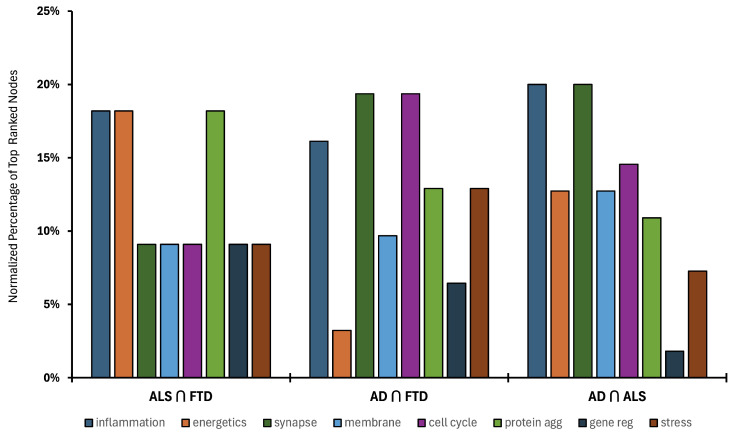
Bar chart illustrating the normalized biological mapping of the top 1% of high-ranking AAPP nodes adjusted for differences in node count between intersecting diseases: AD ∩ ALS, AD ∩ FTD, and ALS ∩ FTD.

Next, the biological mapping classifications obtained from the top-ranking intersecting disease nodes were normalized for differences in overall node count for each disease pair intersection. The normalized percentage of top-ranked nodes for each mapped biological process is shown for each of the three disease intersections in Figure 5. The normalization allows the percentage contributions of each category of biological processes to be equivalently compared for each disease intersection. The percentages range from 2% to 20% across the eight biological processes. The intersection of ALS and FTD was predominated by inflammation and immune response, energetics, and protein aggregation. The intersections of AD with FTD and AD with ALS were predominated by synapse and neurotransmission and inflammation and immune response. Notably, inflammation and immune response was the primary biological process that all disease intersections shared.

**Figure 6:**
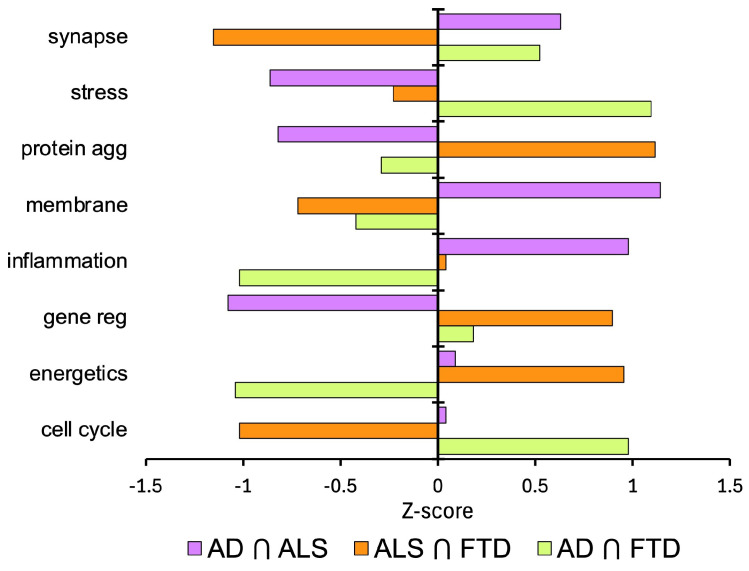
Z-scores to examine standardized differences in the biological process mappings of disease intersections compared to the overall average for each biological process: AD ∩ ALS, ALS ∩ FTD, and AD ∩ FTD.

Finally, the Z-scores are calculated in Figure 6 to examine standardized differences in the biological process mappings of disease intersections compared to the overall average for each biological process. A Z-score is interpreted based on its sign and magnitude: a positive Z-score indicates the data point is above the mean for that biological process, and a negative Z-score indicates it is below the mean for that biological process. The absolute value of the Z-score indicates the number of standard deviations it is away from the overall mean for that biological process. A larger absolute value of the Z-score means it is further from the mean.

Based on the positive Z-scores in Figure 6, the intersection of AD and FTD was substantially higher than the mean for cell cycle regulation and stress response. The intersection of ALS and FTD was substantially higher than the mean for protein aggregation, gene regulation, and energetics. The intersection of AD and ALS was substantially higher than the mean for synapse and neurotransmission, membrane regulation, and inflammation and immune response.

### 2.4. Examination of Overlapping Genetics

Genetic overlap between AD, ALS, FTD, and co-occurring ALS-FTD, AD-FTD, and ALS-AD has suggested the possibility of a disease spectrum. Minimally, it indicates the presence of shared genetic susceptibility to neurodegeneration. Table 3 lists the genes and proteins that overlap in AD, ALS and FTD with the corresponding HeteSim scores for each disease. The HeteSim score is a relevance score that indicates the listed gene’s overall relevance or importance to the target node (the specified disease) as determined by the SemNet 2.0 unsupervised learning ranking algorithm [21]. Scores closer to 1 represent higher relevance to the target disease.

The hexanucleotide repeat expansion *C9orf72* has been detected in a portion of cases presenting with ALS, FTD, the combined ALS-FTD condition [25], AD, and the combined AD-FTD condition, among others [26]. Additionally, a wide spectrum of proteins and genes have been found to be associated with FTD, ALS, and FTD-ALS, including microtubule associate protein TAU gene (MAPT) [27], progranulin (GRN) [28], Valosin-containing protein gene (VCP) [29], charged multivesicular body protein 2B gene (CHMP2B) [30], superoxide dismutase 1 gene (SOD1) [31], TAR DNA binding protein gene (TARDBP or TDP-43) [32], and fused in sarcoma gene (FUS) [33]. However, these genes were also found to be related to AD. For example, *C9orf72* repeat expansions have been detected in several cases of familial AD [34]. MAPT and GRN have also been reported in clinical AD cases [35,36].

### 2.5. Top-Ranked Overlapping AAPP Nodes in AD, ALS, and FTD

Table 4 lists the names of the source nodes and the composite scores shared between two diseases. The composite score is determined in CompositeView [24] by computing the arithmetic mean of the HeteSim scores for all outgoing edges linked to each source node. Any two diseases formed by a combination of AD, ALS, and FTD are called disease pairs. Thus, there are three distinct disease pairs: AD ∩ FTD, AD ∩ ALS, and ALS ∩ FTD.

#### 2.5.1. Top-Ranked Nodes at the Intersection of AD and FTD

Among the 1% of top-ranked AAPP nodes for the intersection of AD and FTD are the following from Table 4:

- Phosphatidylserine (PS) is a membrane phospholipid essential for signaling, apoptosis, and membrane fluidity, particularly in neurons. Dysregulated PS metabolism impairs mitochondrial integrity, energy production, and immune clearance, exacerbating neurodegeneration [37]. PS also interacts with misfolded proteins, such as amyloid-beta and TDP-43, affecting their aggregation and toxicity.
- Homer 1a, a neuronal synaptic protein, modulates mGluR signaling, calcium homeostasis, and synaptic plasticity. Its reduction contributes to cognitive decline and memory loss by disrupting synaptic protein balance and impacting synpatic plasiticy [38]. Homer 1a dynamically responds to cellular stress and influences protein aggregation pathways involving amyloid-beta and TDP-43.
- Syntaxin 3B, a SNARE protein, facilitates vesicle docking, fusion, and neurotransmitter release. Its dysfunction in AD, ALS, and FTD disrupts synaptic signaling, leading to cognitive and behavioral symptoms [38]. Syntaxin 3B also supports vesicle trafficking, and its impairment under cellular stress promotes protein aggregation and defective homeostasis.
- The EphA3 receptor governs neuronal development, axonal guidance, and synaptic plasticity. Dysregulated EphA3 signaling contributes to synaptic loss, chronic neuroinflammation, and impaired connectivity that is modulated via the interaction of nicotinic receptors in areas like the hippocampus [39]. EphA3 also likely influences cell survival and apoptosis pathways and interacts with protein aggregates like amyloid-beta and TDP-43.
- CDK11B (Cyclin-Dependent Kinase 11B) regulates the cell cycle, transcription, and apoptosis. Aberrant CDK11B activity in neurodegeneration drives inappropriate cell cycle re-entry, neuronal apoptosis, and stress responses, increasing neuronal vulnerability and promoting protein aggregation, especially in AD [40].
- Phospholipid serine base exchange enzyme controls PS metabolism, a key regulator of apoptosis, mitochondrial integrity, and immune activation. Dysregulated PS synthesis triggers neuroinflammation, oxidative stress, and excessive neuronal clearance by immune cells [37].
- The HOXC gene cluster, part of the homeobox family, is critical for neuronal differentiation and maintenance [41]. Dysregulated HOXC expression in neurodegenerative diseases disrupts neuronal survival and may exacerbate inflammation.
- TCF4 and TCF7L2, transcription factors in the Wnt signaling pathway, modulate synaptic plasticity, immune responses, and inflammation. Genetic variants increase susceptibility to neurodegeneration, cognitive decline, and metabolic disorders.
- Thyroid hormone receptor alpha-1 (THRA1) regulates neuronal energy metabolism, synaptic plasticity, and antioxidant defenses [42]. Thyroid dysfunction leads to energy deficits, oxidative stress, and impaired synaptic function, contributing to neurodegeneration, and has been shown to be a risk factor for AD [43].

#### 2.5.2. Top-Ranked Nodes at the Intersection of AD and FTD

Interestingly, the CompositeView in Figure 2 shows only a few top-ranking nodes at the intersection of ALS and FTD. This finding was unexpected given that the combined ALS-FTD clinical presentation is the most discussed and frequent co-occurring clinical diagnosis among AD-FTD, AD-ALS, and ALS-FTD. Similarly, Table 4 shows that the intersection of ALS and FTD had 43,368 nodes. However, the CompositeView filters to create a subset of the most important nodes and aggregates their HeteSim scores into a composite score. Once this process was completed, a tractable number of important intersecting nodes remained. Among the top 1% of AAPP nodes for the intersection of AD and FTD are the following from Table 4:

- MicroRNA-150 (miR-150) regulates gene expression in neuronal survival, influencing neuroinflammation and neurodegenerative processes. Upregulated blood miR-150-5p in Alzheimer’s Disease has been associated with cognition, cerebrospinal fluid amyloid-β, and cerebral atrophy [44]. Other similar microRNA biomarkers have been proposed to distinguish AD and ALS [45].
- SUGT1 (Suppressor of G2 allele of Skp1 homolog 1) is a protein involved in cell cycle regulation, autophagy, and proteasome function, which protects neurons from oxidative stress [46]. Its dysfunction in ALS and FTD promotes protein aggregation and accelerates neuronal death, which is likely enhanced through an association with *C9orf7* [46].

#### 2.5.3. Top-Ranked Nodes at the Intersection of AD and ALS

Among the 1% of top-ranked AAPP nodes for the intersection of AD and FTD with direct evidence are the following from Table 4:

- Bradykinin B2 receptor (B2R) mediates inflammation, vascular permeability, and amyloid-beta metabolism. Dysregulated B2R activity in AD and FTD worsens neuroinflammation, blood–brain barrier dysfunction, and amyloid pathology [47].
- Toll-like receptor 5 (TLR5) links gut microbiome dysbiosis to neuroinflammation in AD and FTD. It interacts with amyloid-beta and tau aggregates, amplifying innate immune responses and contributing to disease progression [48].
- Nuclear matrix binding proteins (NMBPs) maintain a nuclear structure and RNA transport. Dysregulated NMBP function in AD impairs transcriptional regulation, RNA-binding protein dynamics, and tau or TDP-43 metabolism, driving neurodegeneration [49].
- TBP-associated factor 15 kDa (TAF8), also known as IGFBP7, regulates apoptosis, mitochondrial function, and inflammation [50]. Dysregulation of TAF8 contributes to oxidative stress, immune activation, and neuronal loss.
- ATP8A2, a P-type ATPase, maintains lipid homeostasis, mitochondrial function, and calcium dynamics. Dysfunction in ATP8A2 disrupts neuronal energy production and promotes oxidative stress, contributing to the pathogenesis of ALS, AD, and FTD. Prior work has shown its direct ties to axonal degeneration [51].
- Fibrosin relationships are mostly in the context of wound healing, as it regulates extracellular matrix remodeling [52] and is thought to play a role in glial scar formation. Dysregulated fibrosin in AD and ALS could increase inflammation and oxidative stress, contributing to neuronal damage.
- Cytosolic thyroid hormone-binding delivers thyroid hormones, T3 and T4, to the nuclear thyroid hormone receptors. It is hypothesized that disturbances in thyroid hormone levels, particularly reduced levels of T3, may contribute to the cognitive impairments seen in AD. Reduced thyroid hormone activity could also contribute to motor neuron vulnerability, as these neurons rely on appropriate thyroid hormone signaling for survival [42].
- Type I interferon receptor|AVP|PPOX—The Type I interferon receptor is a critical component of the immune response, inflammation, and cellular responses in the central nervous system. Interferons increase pro-inflammatory cytokines in astrocytes and microglia [53]. The related protoporphyrinogen oxidase (PPOX) is an enzyme involved in heme biosynthesis. Disturbances in heme biosynthesis likely contribute to oxidative stress or cellular dysfunction in neurodegenerative diseases.
- SLC6A7 uptakes proline from the extracellular space into neurons and other cells, which plays a critical role in protein synthesis, cellular metabolism, and neurotransmission. Proline is also key to glutamate synthesis [54]. Glutamate is the main excitatory neurotransmitter in the brain, and its dysfunction can lead to excitotoxicity, a process that contributes to neuronal damage and degeneration seen especially in ALS but also in AD and FTD.
- Substance P|CHN1|NEK9—the combination of Substance P, CHN1 (also known as Chimerin 1), and NEK9 (NIMA-related kinase 9) involves a complex interplay of proteins that have distinct roles in neurotransmission, cell signaling, and neuroinflammation. The NK1 receptor has been identified as a potential target for managing inflammation and excitotoxicity in ALS. NEK9 is tied to microtubule stability dynamics [55], potentially impacting tau in AD, FTD, and axonal transport deficiencies in ALS.
- ATP synthetase complexes—specifically ATP8A2, which is a P-type ATPase, is critical for the function of mitochondria, the energy-producing organelles within cells. ATP8A2, in particular, is involved in maintaining the lipid composition of cell membranes, especially in neurons [56]. ATP8A2 is tied to mitochondrial energy production, membrane lipid homeostasis, and calcium homeostasis, which are all known to contribute to the AD, ALS, and FTD etiologies.

#### 2.5.4. Determination of Indirect Nodes

The top 1% of SemNet 2.0 nodes contained some nodes for which there was only indirect evidence for their involvement in the molecular pathophysiology for the disease intersection of interest. This was expected given that the SemNet 2.0 parameters intentionally allowed the inclusion of longer metapaths to better incorporate cross-domain knowledge into the relevance rankings. The type of evidence, direct or indirect, was evaluated using SemNet 2.0, LLM (GPT-4o), and human evaluators as shown in Table 5. Full-text evaluation of data sources by human evaluators served as the ground truth.

Recall AD and ALS had the most intersecting nodes, as shown in the CompositeView layout in Figure 2. Such nodes often were tied to the importance of the gut microbiome, namely its role in neuroinflammation, specific ion channels, and synaptic transmission. Examples of nodes with indirect evidence are detailed below:

- The AL1 protein, tomato golden mosaic virus is a plant virus protein involved in plant cell replication and apoptosis [57]. While viral proteins in general can affect cellular pathways involved in neurodegeneration, inflammation, and protein aggregation, the AL1 protein from TGMV has not yet been directly linked in humans. Study results contend that this node provides indirect evidence for a tie-in to neuorinflammation via the gut microbiome.
- Xylanase X22 is a plant cell wall-degrading enzyme. Indirect mechanisms involving the gut–brain axis, microbial metabolism, and neuroinflammation could suggest potential roles for microbial enzymes like Xylanase X22 in modulating disease processes, likely via dietary fiber breakdown and immune system modulation [58].
- Polyketide Synthase WA produces microbial-derived compounds. Polyketides can be produced by bacteria, fungi, and plants and have diverse biological activities that range from antimicrobial and anticancer effects to immune modulating responses, inflammation, and oxidative stress [59]. Dysregulated polyketides could potentially influence neuroinflammation via the gut–brain axis.
- N-(4-isothiocyano-2-nitrophenyl)-2-aminoethanesulfonate is a chemical compound used in biochemical research to investigate various aspects of cellular function, particularly in the context of membrane transport and ion channel activity.
- Cobatoxin2, cobatoxin 1, and the related protein carassin, are toxins derived from the venom of the Conus genus, particularly marine cone snails. Cobatoxin, like other conotoxins, has potential neurotoxic effects by interacting with voltage-gated ion channels, specifically sodium (Na+) channels [60], and potentially other receptors involved in neurotransmission. Sodium channels have been particuarly documented as important in ALS’s pathophysiology [61].
- Omega-conotoxin RVIA, conus radiatus Omega-conotoxin RVIA (also known as ω-conotoxin RVIA) is a potent peptide toxin derived from the venom of the marine cone snail species conus radiatus. This toxin selectively targets N-type voltage-gated calcium channels (Cav2.2), which are primarily involved in the release of neurotransmitters at synapses [62]. Its high ranking is an indirect link magnifying the importance of calcium signaling in neurotransmission and homeostasis.
- Menchyme Forkhead 1 (FOXP1) is a member of the forkhead box (FOX) family of transcription factors, which play a crucial role in regulating the expression of genes involved in various cellular processes, including cell development, differentiation, and function. They are also implicated in cancers [63]. Multiple cancer-related genes have been previously associated in omics research examining AD [9].

### 2.6. High-Ranking Nodes Exclusive to a Single Disease

Table 6 lists some of the nodes which only belong to one disease within the 99% percentile (e.g., top-ranked 1% of AAPP source nodes). Since there is only one edge connecting source nodes to the target nodes, the composite score is the same as the HeteSim score. Examples of exclusive high-ranking nodes are detailed below:

- The AKAP10 gene regulates cAMP signaling, and cAMP has been shown to be critical in neurodegeneration [64].
- The UGA4 protein is a protein in yeast that is involved in the utilization of gamma-aminobutyrate (GABA) as a nitrogen source. There is no direct evidence for its role in humans, but its ranking indirectly indicates the role of GABA in AD.
- Thrombin|CALR is involved in inflammation and coagulation and has been implicated in AD [65].
- LCP1 (Lymphocyte Cytosolic Protein 1) is mostly tied to cancers, but its ranking indirectly indicates a role for inflammation.
- PGEs (Prostaglandins E), a major product of COX-2 activity, have been reported in patients with probable AD, and are likely tied to inflammation [66].
- Secretory phospholipase A2 is involved in increased inflammatory and decreased metabolic processes [67], which are both seen in AD.
- Fatty-acid peroxidase imbalances may influence neurodegeneration, which is linked to ALS and AD.
- MAPs (microtubule-associated proteins), and namely dysregulated MAPs, such as tau, are hallmarks of AD [68].
- KCNC4 (Potassium Channel Kv3.4) disregulation has been implicated in hyperexcitability in ALS [69].
- FN1 (Fibronectin 1) is associated with extracellular matrix remodeling [70], which is known to be a relevant factor in ALS.
- GEMIN8 is part of the survival of motorneuron (SMN) complex [70], which makes it relevant to ALS.
- FGFBP3 (Fibroblast Growth Factor Binding Protein 3) is involved in growth signaling, as well as fat and metabolic signaling. The role of metabolism in the molecular pathophsyiology of AD is well documented [9].
- Cholinergic receptors in ALS are involved in modulating motor neuron activity. Dysfunction of these receptors, particularly nicotinic acetylcholine receptors (nAChRs) at the neuromuscular junction, contributes to the impaired signal transmission, muscle weakness, and motor neuron degeneration seen in ALS [71].

### 2.7. Do AD, FTD, and ALS Lie on the Same Spectrum?

AD, FTD, and ALS share a common subset of underlying symptoms, mechanisms, and risk factors. The overlapping features of these neurodegenerative diseases may indicate a pathophysiological spectrum. The presence of disease overlap has important implications for diagnosis, treatment, and prevention strategies. It suggests that proactive interventions or modalities targeting common underlying factors may be beneficial for multiple conditions. Additionally, it underscores the importance of considering the broader context of neurological disease processes rather than viewing each as a siloed condition.

The link between ALS and FTD has been widely acknowledged, with recent data-based evidence revealing what appears to be a spectrum of disease even in brain-MRI patient sub-typing [72]. A link between AD and FTD has also been suggested but is not as well established. The diagnosis of a mixed AD-FTD pathology is difficult given both diseases impact the frontal region on MRI. Unlike AD, FTD symptoms may manifest while cognition remains spared [73].

An overarching conclusion of the SemNet 2.0 results is that the top-ranked nodes of FTD tend to have great overlap with both ALS and AD. As such, the results of the present study suggest FTD to have the least differentiated pathology compared to AD and ALS. This might explain why mixed FTD etiologies have been cited within ALS [72], AD [73], and even Parkinson’s Disease [74].

While AD and ALS also had a high degree of overlap in the present study, their functional overlap in individual clinical studies is not as commonly acknowledged as ALS-FTD or AD-FTD. It appears that many of the same fundamental AAPPs may be involved in the underlying etiologies of AD and ALS but with different functional effector cells or differently impacted functional regulation. Such differences could explain their different functional phenotypes.

In summary, the pathologies of AD, ALS, and FTD share many of the same nodes and edges in the investigated network of cross-domain literature relationships from 33+ million journal articles. Further experimental assessment is necessary to see if the molecular pathophysiology networks of ALS, FTD, and AD comprise a disease spectrum where different forms of dysregulation result in different functional phenotypes [75].

### 2.8. Limitations

The most important limitation is that the SemNet 2.0 algorithm is unsupervised. Immediate experimental or clinical testing of thousands of top-ranked relationships is not feasible, although the work presented helps to prioritize such future studies. Notably, prior work has illustrated that SemNet predictions early in the COVID-19 pandemic [76] translated well to real-world clinical trials for drug repurposing [77]. Additional works utilizing SemNet 2.0 to predict long-term chemotherapy adverse events [23] or underlying mechanisms of disease [78] showed utility for prioritizing drug repurposing and research or experimental hypotheses, including for neurological diseases [22]. Finally, to minimize the limitations of SemNet 2.0, this study also uniquely employed large language models and supervised human evaluation of full-text articles to verify the existence of the SemNet 2.0 knowledge graph relationships and to determine if the evidence provided was a direct or indirect link to the target disease(s) of AD, ALS, or FTD.

## 3. Materials and Methods

**Figure 7:**
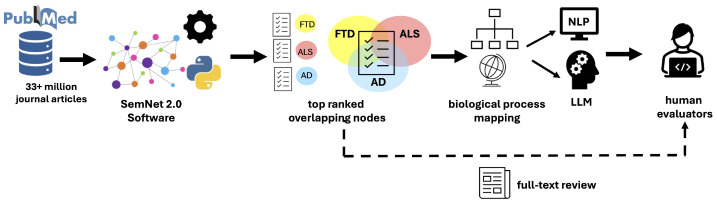
Overall framework for artificial intelligence-based comparative analysis of the molecular pathophysiology overlap of AD, ALS, and FTD. Over 33 million journal articles from PubMed were used to construct the knowlege graph used by SemNet 2.0. The open-source SemNet 2.0 software [21] and its post-visualization software, CompositeView [24], were used to determine which Unified Medical Lanaguge System (UMLS) amino acid, peptide, and protein (AAPP) nodes are most important to AD, ALS, FTD, and, namely, their intersections. Next, the top 1% of nodes were mapped to eight biological processes using cross-domain text mining natural language processing and a large language model. Finally, three human evaluators provided a check on the top 1% of nodes via full text article review and on the corresponding biological mappings.

This study combines both unsupervised and supervised artificial intelligence approaches to examine the overlapping molecular physiology of AD, ALS, and FTD. SemNet 2.0 [21] was utilized to query a knowledge graph of biomedical concepts related to AD, FTD, and ALS in different domains to determine high-ranking biomedical concepts. High-ranking nodes in the Unified Medical Language System (UMLS) node type of amino acids, peptides, and proteins (AAPPs) were evaluated using CompositeView [24], a special graph-based visualizer that better enables simultaneous aggregation, analysis, and visualization of multiple SemNet 2.0 simulations. The highest ranking concepts that were either overlapping or were specifically distinct to AD, FTD, or ALS are reported, visualized, and analyzed to better quantify to what extent and to see if the underlying etiologies potentially overlap. Two different models were used to map individual nodes to broader biological processes: a standard natural language processing [23] and a large language model (LLM). Finally, supervised human-in-the-loop validation was performed as part of model evaluation. This framework is shown in Figure 7.

### 3.1. Overview of SemNet 2.0

SemNet 2.0 is a Python-based open-source software designed to interact with a biomedical knowledge graph (KG) comprising semantic triples extracted from PubMed’s extensive data-base of over 33 million abstracts [21]. Each semantic triple within this knowledge graph contains a head, relation, and tail. The head and tail entities serve as nodes, and the relation is represented as a directed edge. These nodes correspond to biomedical concepts categorized within the United Medical Language System (UMLS), which has 133 node types and 54 relations. The directed edges encapsulate relations like ‘treats’, ‘affects’, ‘inhibits’, etc. SemNet 2.0 uses an unsupervised ranking algorithm to determine to rank the most relevant or “important” nodes to a given target node input.

#### 3.1.1. Search Parameters

There are four user-defined inputs: the target nodes, source node types, search depth, and metapath length. Target nodes are defined as the primary nodes of the interest, which, in this study, are AD, ALS, and FTD. The search node types are semantic types specified by the UMLS ontology. Search depth is defined as the number of connections or “hops” between the target node and the source node. When targeting a specific node *T*, a search depth of 1 encompasses all directly connected adjacent nodes. Increasing the search depth is advantageous for discovering new findings. Connections between neighbors and target nodes are more readily apparent and widely acknowledged in the scientific literature. Metapath length, on the other hand, represents the cumulative distance traveled from a target to a source node. Multiple pathways can be condensed into a single metapath based on the types of source nodes involved.

Unique UMLS IDs and corresponding concept unique identifiers (CUIs) were assigned to the three diseases, Alzheimer’s Disease (CUI: C0002395), Amyotrophic Lateral Sclerosis (CUI: C0002736), and Frontotemporal Dementia (CUI: C0338451). The amino acid peptide protein (AAPP) was chosen for each disease type as it best represents the sub-molecular and molecular pathophysiological relationships. The search depth was 2 and the metapath length was 3 for all SemNet 2.0 simulations. The parameters for this application-based molecular relationships study were chosen based on optimizations computed as part of previous theoretical machine learning research [21].

#### 3.1.2. Definition of HeteSim Score

SemNet 2.0 employs a metric known as HeteSim to evaluate the relatedness or relevance between a source node and a target node [21]. HeteSim is a similarity metric that compares metapaths that connect to the user-defined target node(s). The comparing of metapaths via the calculation of a HeteSim score enables the relative importance to the target node to be quantified. This study utilized the deterministic HeteSim setting and the exact mean for aggregating HeteSim scores from multiple target nodes to the same source node.

#### 3.1.3. Normalization of HeteSim Scores

HeteSim scores can vary as a function of simulation parameters and number of returned nodes. Therefore, HeteSim scores are only comparable within individual simulations. Since cross-domain text mining involves multiple simulations, normalization is necessary to ensure fair comparison across multiple SemNet 2.0 simulations performed for different disease(s). Consequently, the HeteSim results used for ranking relevant source nodes were normalized by removing the mean, scaling to unit variance, and percentile ranking. A percentile ranking ensured equivalency to fairly compare and contrast node rankings from different simulations.

#### 3.1.4. CompositeView

CompositeView is a visualization tool that was customized to best visualize and perform post hoc analysis on SemNet 2.0 output [24]. CompositeView visualizes the high-ranked related source nodes and calculates composite scores. Collectively, CompositeView enables the resultant graph resolution to be increased or decreased by combining and averaging HeteSim scores. In the case of SemNet 2.0, the composite score is determined by computing the arithmetic mean of all outgoing edges linked to each source node. This composite score combines information into a unified metric that, while less detailed, offers insights from the data in a manner that justifies the trade-off of minimal information loss. The composite score associated with each source node reflects its relevance to AD, ALS and FTD.

#### 3.1.5. Human-in-the-Loop Supervised Validation of High-Ranking Nodes

SemNet 2.0 utilizes an unsupervised learning rank aggregation algorithm to output which source nodes are most related to the target diseases [21]. The unsupervised algorithm has no ground truth with which to directly compare the output and evaluate model performance. As such, a supervised human-in-the-loop process was used to manually check high-ranking source nodes by evaluating the full-text articles from which the high-ranking source node relationships were extracted. Evaluators examined the full-text article to determine the presence of either direct or indirect evidence to support node relevance to AD, FTD, or ALS. Reviewed full-text articles were selected using the PubMed Unique Identifier (PMID) of articles used to construct the relations in the knowledge graph. Human evaluation provided both a supervised check on the knowledge graph and the ranking algorithm.

The evidence for the top-ranked 1% of SemNet nodes was also classified as either direct or indirect. Classification of direct or indirect by SemNet was based on the shortest metapath link to the target node(s). Classification by the GPT-4o LLM used a binary classification prompt using the top-ranked SemNet 2.0 nodes as a .csv input. Classification by humans (used as ground truth) was based on the full-text evaluation described above. Human evaluation served as the ground truth for the precision, recall, F-measure, and accuracy shown in Table 5.

### 3.2. Post-Simulation Mapping of Biological Processes

Molecular and proteomic disease research often maps molecules to biological processes to improve explainability and comprehension [9]. The top 1% of high-ranking AAPP source nodes were assigned to one or more defined biological processes using post-simulation SemNet 2.0 output.

### 3.3. Statistical Analysis of Biological Process Overlap

Z-score analysis for each biological process was performed to examine relative differences in the prevalence of biological processes at disease intersections. Z-scores were calculated and visualized in Microsoft Excel.

#### 3.3.1. Definitions of Mapped Biological Processes

Relevant study-specific definitions of biological processes were honed by combining the UMLS ontology and natural language processing using supervised domain expertise, as has been conducted in previous cross-domain text mining works [23,79]:

- Cell cycle regulation: nodes involved in the cell cycle, including mitosis and apoptosis, as well as tumor factors or erratic signaling typically associated with neoplasms.
- Energy and metabolism: nodes involved in cellular respiration, glucose metabolism, lipid metabolism, energy conversion, metabolic regulation, redox reactions that contribute to free radicals or oxidative stress, and all other processes encapsulated in the mitochondria and their regulation.
- Gene regulation and expression: nodes that direct, modify, or otherwise regulate the expression of one or more genes. Gene expression may include physiological gene expression, dysfunctional gene expression, non-cancerous mutated gene expression, DNA repair, DNA replication, and transcriptional regulation.
- Inflammation and immune response: nodes involved that promote, inhibit, or provide other regulate inflammatory or immune responses. This can include local neuroinflammation from the microglia and astrocytes in response to the release of stimulatory cytokines or neurotransmitters in their microenvironment or it could be systemic inflammation; autoimmune inflammatory processes; immune responses to external, infectious, or environmental pathogens; or stimuli.
- Membrane regulation: nodes related to membrane regulation, membrane lipid homeostasis, transport of molecules into and out of the cell and/or cell membrane, the expression of receptors in the membrane, etc.
- Protein aggregation: nodes related to the translation, destruction, and overall regulation of proteins, namely misfolded proteins that lead to plaques, tangles, etc.
- Stress response regulation: nodes that are involved in the regulation of cellular or systemic stress, including the hypothalamic–adrenal–pituitary axis, thyroid, etc., and the production, release, modulation, or regulation of stress-reducing or modulating molecules or hormones and/or their effector cells, tissues, or organs.
- Synapse and neurotransmission: nodes that are involved in excitation, inhibition, or other regulation of signaling neuron(s), network regulation or synaptic plasticity, or the development and maintenance of physical neural structures. This also includes neurotransmitters, neuromodulators, and ion channels (e.g., potassium channels, calcium channels, etc.).

#### 3.3.2. Model Classification of High-Ranking Nodes to Biological Processes

The text of the .csv SemNet 2.0 output files were processed to assign biological mappings. Two existing methods were adapted for this task: standardized post hoc natural language processing [23] and large language models [19].

First, natural language processing was used to map returned AAPP source nodes to one or more biological processes using UMLS ontology mapping. This mapping did not explicitly specify a tie to AD, FTD, or ALS. However, the AD, FTD, and ALS relationships were inferred from SemNet metapaths that illustrate relationships between each identified source node and its target node (i.e., AD, ALS, or FTD).

Second, an external large language model, GPT-4o, was used to map the source nodes in the .csv file. The prompt specifically asked GPT to return from the list of biological processes which mappings were most specific to AD, FTD, or ALS. If the LLM did not have mappings due to a lack of direct connection to AD, FTD, or ALS, it was prompted to state what the connection of the input nodes may be. This last prompt was primarily used to help in supervised human validation of the model(s) to better understand discrepancies in mappings.

#### 3.3.3. Supervised Validation of Biological Mappings

Three trained human evaluators examined the biological mapping classifications returned by the NLP and LLM algorithms. In particular, human evaluators examined mappings where model results did not intersect. The NLP and LLM algorithms did agree on all mappings where there was direct evidence. However, the LLM did not return all relevant mappings when there was no overt or direct connection between the nodes and ALS, FTD, or ALS. The separate prompt requesting the LLM to state what the connection might be provided human evaluators context for possible model error evaluation.

## 4. Conclusions

This study demonstrated that artificial intelligence models are a comprehensive and attractive method to identify, compare, and contrast the multi-factorial molecular pathophysiology of overlapping neurological diseases using data from 33+ million biomedical journal articles. FTD shared 99.9% of its AAPP nodes with ALS and AD; AD shared 64.2% of its AAPP nodes with FTD and ALS; and ALS shared 68.3% of its AAPP nodes with AD and FTD. The overall percentage that each intersecting biological process comprised was as follows: inflammation and immune response, 19%; synapse and neurotransmission, 19%; cell cycle, 15%; protein aggregation, 12%; membrane regulation, 11%; stress response and regulation, 9%; gene regulation, 4%. The presented SemNet 2.0 node rankings for AD, ALS, and FTD provide an objective, evidence-based format to prioritize future research, drug targets, and diagnostic risk factor identification. The results illustrate that AD, ALS, and FTD share a large degree of underlying network relationship dynamics and likely comprise a multi-factorial neuropathological spectrum. Small differences in the pathophysiological network composition or its regulation likely shape the underlying expressed disease phenotype.

## Figures and Tables

**Table 1:** Summary counts of returned source nodes from each SemNet 2.0 simulation. Target nodes are the diseases of interest: AD, ALS, and FTD. Source node type was limited to the Unified Medical Language System (UMLS) node type of amino acid, peptide, and proteins (AAPP). The number of returned source nodes from each SemNet 2.0 simulation [21] is recorded.

Disease	AAPP
AD	67,213
FTD	43,643
ALS	63,179

**Table 2:** Summary of the number of AAAP source nodes shared by AD, ALS, and FTD as determined by SemNet 2.0 [21]. The intersection is shown for each of the three disease pairs and for all three diseases.

Disease	AAPP
AD∩FTD	43,418
AD∩ALS	62,335
ALS∩FTD	43,368
ALL	43,176

**Table 3:** A list of some common genes and proteins shared by AD, ALS, and/or FTD. Each gene or protein has its HeteSim score shown for each disease. The exception is superoxide dismutase 1 protein (SOD1), which is only shared by ALS and FTD.

Names	AD	ALS	FTD
C9orf72 protein|C9orf72	0.794	0.783	0.548
C9orf72 gene|C9orf72	0.826	0.823	0.577
MAPT protein, human|MAPT	0.808	0.601	0.407
MAPT gene|MAPT	0.846	0.76	0.673
GRN protein, human|GRN	0.888	0.934	0.988
GRN gene|GRN	0.75	0.757	0.655
TDP-43 protein, human|TARDBP	0.943	0.887	0.65
TARDBP gene|TARDBP	0.803	0.716	0.552
RNA-binding protein FUS	0.933	0.936	0.956
FUS gene|FUS	0.768	0.609	0.59
superoxide dismutase 1|SOD1		0.831	0.639
SOD1 gene|SOD1	0.672	0.701	0.725
VCP protein, human|VCP	0.91	0.969	0.942
VCP gene|VCP	0.695	0.865	0.713
CHMP2B gene|CHMP2B	0.625	0.912	0.827

**Table 4:** A list of some nodes shared by different disease pairs for AAPP node type. Node names are exactly returned from the SemNet simulations and composite scores are calculated in CompositeView by computing the arithmetic mean of two HeteSim scores from the disease pair [24]. All the intersecting nodes with FTD are listed. However, due to space limitations, only the top 20 nodes (based on composite score) are shown for the intersection of AD and ALS.

Node Names	Composite Scores	Intersections
Phosphatidylserine decarboxylase 2	0.87	AD ∩ FTD
Homer 1a	0.513	AD ∩ FTD
Syntaxin 3B	0.476	AD ∩ FTD
EphA3 receptor	0.141	AD ∩ FTD
CDK11B	0.704	AD ∩ FTD
Phospholipid serine base exchange enzyme	0.461	AD ∩ FTD
HOXC@ gene cluster	0.935	AD ∩ FTD
TCF4|TCF7L2	0.911	AD ∩ FTD
Thyroid hormone receptor alpha-1	0.753	AD ∩ FTD
MIR150	0.947	ALS ∩ FTD
SUGT1	0.986	ALS ∩ FTD
AL1 protein, tomato golden mosaic virus	0.998	AD ∩ ALS
Bradykinin B2 receptor|KNG1	0.998	AD ∩ ALS
TLR5 protein, human|TLR5	0.998	AD ∩ ALS
Nuclear matrix binding proteins	0.998	AD ∩ ALS
TBP-associated factor 15 kDa|IGFBP7|TAF8	0.985	AD ∩ ALS
Xylanase X22	0.998	AD ∩ ALS
Polyketide synthase WA	0.998	AD ∩ ALS
Fibrosin	0.996	AD ∩ ALS
Omega-conotoxin RVIA, conus radiatus	0.998	AD ∩ ALS
Cytosolic thyroid hormone-binding protein, Xenopus	0.998	AD ∩ ALS
Type I interferon receptor|AVP|PPOX	0.998	AD ∩ ALS
Cobatoxin 1	0.998	AD ∩ ALS
Alpha-conotoxin PnIB	0.998	AD ∩ ALS
N-(4-isothiocyano-2-nitrophenyl)-2-aminoethanesulfonate	0.998	AD ∩ ALS
Glycoproteins|SLC6A7	0.998	AD ∩ ALS
Cobatoxin 2	0.998	AD ∩ ALS
Carassin	0.998	AD ∩ ALS
Mesenchyme fork head 1 protein|FOXP1	0.998	AD ∩ ALS
Substance P|CHN1|NEK9	0.998	AD ∩ ALS
ATP synthetase complexes|ATP8A2	0.998	AD ∩ ALS

**Table 5:** Classification of direct versus indirect evidence for the top-ranked 1% of SemNet 2.0 source nodes. Evidence classification was performed by SemNet 2.0, GPT-4o LLM, and humans. Classification of direct or indirect by SemNet was based on the shortest metapath link to the target node(s). Classification by GTP-4o LLM used a binary classification prompt. Classification by humans was based on full-text evaluation of articles from SemNet 2.0 using PMIDs derived from the knowledge graph. Classification by humans was considered the ground truth, which by definition, resulted in perfect precision, recall, F-measure, and accuracy.

Evaluator	Precision	Recall	F-Measure	Accuracy
SemNet 2.0	0.94	0.71	0.81	0.74
GPT-4o LLM	0.80	0.83	0.82	0.71
Humans	1.0	1.0	1.0	1.0

**Table 6:** A list of some AAPP source nodes that only belong to one of the three target diseases of AD, FTD, or ALS. The composite score in this case is the same as the normalized HeteSim score. Note that there were no exclusive node(s) for FTD.

Node Names	Composite Score	Disease
AKAP10 gene	0.991	AD
UGA4 protein, S cerevisiae	0.992	AD
Thrombin|CALR	0.991	AD
LCP1 protein, human	0.991	AD
PGE synthase 1, human	0.991	AD
Secretory phospholipase A2	0.99	AD
Fatty-acid peroxidase	0.997	AD
MAP kinase kinase 1	0.999	AD
KCNC4	0.999	AD
FN1 wt allele|FN1	0.999	ALS
GEMIN8	0.999	ALS
FGFBP3 gene|FGFBP3	0.999	ALS
Cholinergic receptors|MECP2|PITX2|REG1CP|RS1	0.999	ALS
SKAP55-related protein|CTBS|PCYT1B	0.999	ALS
Recombinant interleukin-9|IL9	0.995	ALS

## Data Availability

SemNet 2.0 and CompositeView software are open source and available at https://github.com/pathology-dynamics, accessed on 12 December 2024.

## Abbreviations

The following abbreviations are used in this manuscript:

DOAJ	Directory of open access journals.
TLA	Three-letter acronym.
LD	Linear dichroism.
